# Low physical activity is associated with impaired endothelial function in patients with type 2 diabetes and controls after 5 years of follow-up

**DOI:** 10.1186/s12902-021-00857-9

**Published:** 2021-09-18

**Authors:** Jonathan Mathias Baier, Kristian Løkke Funck, Liv Vernstrøm, Esben Laugesen, Per Løgstrup Poulsen

**Affiliations:** Department of Internal Medicine and Endocrinology and Steno Diabetes Center Aarhus, Aarhus University Hospital, Palle Juul-Jensens Boulevard 99, DK-8200 Aarhus N, Denmark

**Keywords:** Endothelial dysfunction, Endothelial function, Type 2 diabetes, Physical activity, Reactive hyperemia index, Peripheral arterial tonometry, EndoPAT, Accelerometery

## Abstract

**Background:**

The long-term association between physical activity and endothelial function has not previously been investigated in patients with type 2 diabetes. Therefore, we aimed to evaluate the relationship between physical activity and endothelial function, assessed by peripheral arterial tonometry, in patients with type 2 diabetes and non-diabetic controls after 5 years of follow-up.

**Methods:**

We included 51 patients with newly diagnosed type 2 diabetes and 53 sex- and age matched controls. Participants underwent baseline clinical characterization including objective measurement of physical activity level using accelerometery. After 5 years of follow-up, participants were re-examined, and endothelial function was assessed as natural logarithm of reactive hyperemia index (lnRHI).

**Results:**

Physical activity at baseline was associated with lnRHI after 5 years of follow-up in both patients with type 2 diabetes and controls. An increase of 1 standard deviation (SD) in daytime physical activity corresponded to a 6.7 % increase in RHI (95 % confidence interval: 1.1;12.5 %, *p* = 0.02). We found no difference in lnRHI between patients with diabetes and controls (0.67 ± 0.29 vs. 0.73 ± 0.31, *p* = 0.28).

**Conclusions:**

Daytime physical activity is associated with endothelial function after 5 years of follow-up in patients with type 2 diabetes and controls.

**Supplementary Information:**

The online version contains supplementary material available at 10.1186/s12902-021-00857-9.

## Introduction

The vascular system in patients with type 2 diabetes is characterized by impaired endothelial function [1, 2]. Endothelial dysfunction precedes overt cardiovascular disease (CVD) [3], and it was recently shown that endothelial dysfunction and type 2 diabetes mellitus synergistically increase cardiovascular risk [4]. Thus, it has been suggested that endothelial dysfunction not only adds to the total amount of risk, but potentiates the effect of other traditional risk factors. Hence, evaluation of endothelial function may add important information in early risk stratification of patients with type 2 diabetes.

Assessment of flow-mediated vasodilation (FMD) of the brachial artery using ultrasound is the most widely used non-invasive method for evaluation of endothelial function [5]. However, peripheral arterial tonometry (PAT) has been suggested as an inexpensive, reproducible and less operator-dependent method for evaluation of endothelial function [6, 7]. The two methods differ in target vasculature, as FMD assesses brachial artery diameter and PAT evaluates finger artery pulse wave. Moreover, The Framingham Heart Study found no significant relationship between the two methods, suggesting that they reflect different characteristics of vascular function [8]. Nonetheless, both FMD and PAT are independent predictors of cardiovascular (CV) events [9]. Specifically, PAT has demonstrated prognostic value as a predictor of CV events in populations with coronary artery disease and heart failure [10–14]. Furthermore, a recent prospective study reported that PAT was able to independently predict CV events in patients with type 2 diabetes and albuminuria [15]. Some [16–21], but not all studies [22–25] have found impaired endothelial function assessed by PAT in patients with diabetes compared with non-diabetic subjects.

Physical activity has long been one of the cornerstones of non-pharmacological treatment of type 2 diabetes. Although the Look AHEAD Trial did not show any effect of intensive lifestyle intervention (including increased physical activity) on major cardiovascular events, the participants in the intervention arm achieved better glycemic control, physical fitness and increased quality of life [26, 27].

As previously reported for this cohort, patients with type 2 diabetes were significantly less physically active compared to controls [28]. A number of short-term interventional studies have shown positive effects of exercise training on endothelial function assessed by FMD in patients with type 2 diabetes [29]. Moreover, observational studies have found that endothelial function is positively associated with high physical activity in healthy adolescents and middle-aged men [30–32]. However, data on the long-term association between physical activity and endothelial function assessed by PAT in patients with diabetes are missing. Therefore, in this prospective study, we aimed to elucidate whether physical activity is associated with endothelial function assessed by PAT after 5 years follow-up in patients with type 2 diabetes.

## Methods

### Subjects

We included participants from a cohort of 100 patients with type 2 diabetes and 100 control subjects [33]. In brief, patients with type 2 diabetes were recruited from the outpatient clinic at Aarhus University Hospital. Control subjects without type 2 diabetes, matched on sex and age, were recruited through advertisement in local press. Enrollment took place between 2009 and 2011 and 5-year follow up examinations were conducted from 2014 to 2016. Inclusion criteria for both groups were age > 18 years; for patients with type 2 diabetes, the diagnosis was confirmed in accordance with recent consensus criteria [34] and diabetes duration was less than 5 years at the time of inclusion. Control subjects underwent diabetes screening with oral glucose tolerance test and fasting glucose and were excluded if diabetes was diagnosed. For both groups, major exclusion criteria were acute or chronic infection, previous or current cancer, pregnancy or lactation. The cohort was established with the incentive of a comprehensive characterization, including physical activity, of patients with newly diagnosed type 2 diabetes compared to healthy controls.

Endothelial function was assessed at the 5-year follow up visit. Participants with complete data on physical activity and endothelial function were included in the present study.

The study protocol was approved by The Central Denmark Region Committees on Health Research Ethics and by the Danish Data Protection Agency. All participants gave their written, informed consent.

### Physical activity

Participants were equipped with an Actiheart combined accelerometer and heart rate monitor (CamNtech), for three consecutive days, while they were instructed to maintain their usual daily activities. The Actiheart accelerometer has a piezoelectric element that is attached to a seismic mass. During acceleration the piezoelectric element will generate an electric charge proportional to the force applied. The data extracted from the accelerometer is expressed as counts per minute (cpm). Mean daytime physical activity was measured as cpm between 6 a.m. and 10 p.m. Examples of moderate to vigorous activities include slow walk (102 cpm), fast walk (597 cpm) and fast running (1908 cpm), whereas low/sedentary activities include computer work (0.3 cpm) and washing dishes (10.6 cpm) [35, 36].

### Endothelial function

Endothelial function was assessed by PAT using the EndoPAT 2000 device (Itamar Medical Inc., Caesarea, Israel). The device consists of a system of inflatable digital probes connected to a computer, which detects digital pulse wave amplitude before, during, and after occlusion of brachial blood flow of the left arm. The right arm serves as control. The examination takes 15 min. After 5 min of baseline measurements, brachial blood flow on the upper left arm is occluded using an inflatable blood pressure cuff (Hokanson SC-12, Bellevue, WA, USA) inflated to 200 mmHg or systolic blood pressure plus 60 mmHg (whichever is higher). The cuff is deflated after 5 min, and the protocol continues for additional 5 min of measurements. The endothelial function is estimated as an index value of the increase in digital pulse amplitude (reactive hyperemia) after deflation of the cuff. The EndoPAT software calculates a Reactive Hyperemia Index-value (RHI), which is a ratio of the PAT amplitude post-to-pre occlusion of the tested arm divided by the post-to-pre occlusion ratio of the control arm. Higher RHI-values reflect better endothelial function. Data are presented as the natural logarithm of RHI (lnRHI) in accordance with manufacturer guidelines. There are no official reference values for lnRHI but values ≤ 0.51 are considered abnormal (Itamar product information). Moreover, Rubinshtein et al. found that values < 0.40 were associated with a higher risk of future CV adverse events [10].

### Other measurements

All participants underwent assessment of anthropometrics, HbA_1C_, lipid profile, urinary albumin:creatinine ratio and ambulatory blood pressure monitoring as well as office blood pressure measurement at baseline and follow-up. Urinary albumin excretion was estimated by urinary albumin:creatinine ratio (UACR) in three morning urine samples. Office BP was assessed as a mean of three consecutive measurements obtained with an oscillometric blood pressure monitor (Riester Champion N; Riester GmbH; Jungingen, Germany) after a minimum of 5 minutes’ rest in seated position. Ambulatory blood pressure was measured every 20 min for 24 h using the Spacelab 90,217 (Spacelabs Healthcare, Issaquah, Washington, USA).

### Statistical analyses

Distribution of data was tested with histograms and QQ-plots. Normally distributed data are presented as mean ± standard deviation (SD), whereas skewed data are presented as median (interquartile range). Categorical values are presented as numbers (%). Means of normally distributed data were compared with Student’s paired *t*-test for paired data, whereas Student’s unpaired *t*-test was used to compare unpaired data. Skewed data were log-transformed in order to obtain normal distribution. If normal distribution was not achieved by log-transformation, a Wilcoxon signed-rank test or Wilcoxon-Mann-Whitney test was applied as appropriate. Dichotomous data were compared using a McNemar’s or chi-squared test. Univariate and multivariate linear regressions were used to evaluate the association between physical activity and lnRHI. *P*-values less than 0.05 were considered statistically significant. Given the exploratory aims of the cohort study, power calculations were not carried out.

Statistical analyses were conducted using Stata 16 (StataCorp LP, College Station, TX, USA).

## Results

### Baseline characteristics

A total of 63 patients with type 2 diabetes and 72 control subjects attended the follow-up visit. Baseline daytime physical activity and lnRHI data was missing in 18 and 13 subjects respectively. Thus, 51 participants with type 2 diabetes and 53 control subjects were included in the analyses. Mean follow-up time was 5.6 ± 0.4 years for patients with type 2 diabetes and 5.4 ± 0.3 years for control subjects. Clinical and biochemical data for patients and controls at baseline are presented in Table 1. Patients with type 2 diabetes had a median diabetes duration of 7.9 years at follow-up. They had good glycemic control and were well-regulated with regard to blood pressure and lipids. Patients with type 2 diabetes did, however, have significantly higher BMI compared to controls. There was no difference between the two groups with regard to smoking status. A larger proportion of patients with type 2 diabetes were treated with antihypertensives, statins and antiplatelet therapy.

**Table 1:** Clinical and biochemical characteristics of the study population. All baseline characteristics are from time of enrolment

Characteristic	T2DM (*n* = 51)	Controls (*n* = 53)	*p*-value
*Clinical*			
Age, years	64.5 ± 9.7	63.2 ± 9.9	0.75
Male sex, n (%)	30 (59)	28 (53)	0.54
Diabetes duration at baseline, years	1.9 (1.0;3.3)	n/a	n/a
24 h blood pressure, mmHg			
Systolic	126 ± 10^a^	124 ± 11^b^	0.84
Diastolic	75 ± 7^a^	75 ± 7^b^	0.46
Office blood pressure, mmHg			
Systolic	127 ± 11^c^	131 ± 15	0.08
Diastolic	80 ± 8^c^	83 ± 10	0.03
Heart rate (beats/minute)	65 ± 10^d^	60 ± 9	0.997
BMI, kg/m^2^	29.9 ± 4.7	26.3 ± 3.8	< 0.001
Smoking status			0.38
Current, *n* (%)	9 (18)	7 (13)	
Former *n* (%)	21 (41)	17 (32)	
Never, *n* (%)	21 (41)	29 (55)	
Previous CVD, *n* (%)	11 (22)	6 (11)	0.12
Daytime physical activity (cpm)	27 ± 15	42 ± 19	< 0.001
*Biochemical*			
HbA_1C_. mmol/mol	48 ± 8	38 ± 4	n/a
HbA_1C_, (%)	6.5 ± 0.7	5.7 ± 0.3	n/a
Total cholesterol, mmol/L	4.3 ± 0.8	5.7 ± 1.0	< 0.001
HDL-C, mmol/L	1.4 ± 0.3	1.7 ± 0.6	< 0.001
LDL-C, mmol/L	2.2 ± 0.7^e^	3.3 ± 1.0	< 0.001
Triglycerides, mmol/L	1.6 (1.0;2.2)	1.2 (0.9;1.6)	0.01
UACR, mg/g	0.46 (0.28;1.04)	0.23 (0.16;0.34)	< 0.001
*Medication*			
Antihypertensive treatment, *n* (%)	34 (67)	15 (28)	< 0.001
Diabetes treatment			
Metformin, *n* (%)	32 (63)	0 (0)	n/a
Sulfonylureas, *n* (%)	6 (12)	0 (0)	n/a
GLP-1 agonist, *n* (%)	0 (0)	0 (0)	n/a
DPP4 inhibitor, *n* (%)	2 (4)	0 (0)	n/a
Insulin, *n* (%)	4 (8)	0 (0)	n/a
Acetylsalicylic acid, *n* (%)	32 (63)	3 (6)	< 0.001
Statin, *n* (%)	38 (75)	10 (19)	< 0.001

^a^*n* = 50

^b^*n* = 52

^c^*n* = 49

^d^*n* = 50

^e^*n* = 49

We found similar clinical and biochemical characteristics in patients with type 2 diabetes that attended follow-up compared with patients with type 2 diabetes that did not attend follow-up or had missing data with the exception that LDL cholesterol was higher in the latter group. Similarly, control subjects that attended follow-up were comparable to control subjects that did not attend follow-up, aside from a lower heart rate, see Supplemental Tables 1 and 2.

### Physical activity and endothelial function

**Fig. 1:**
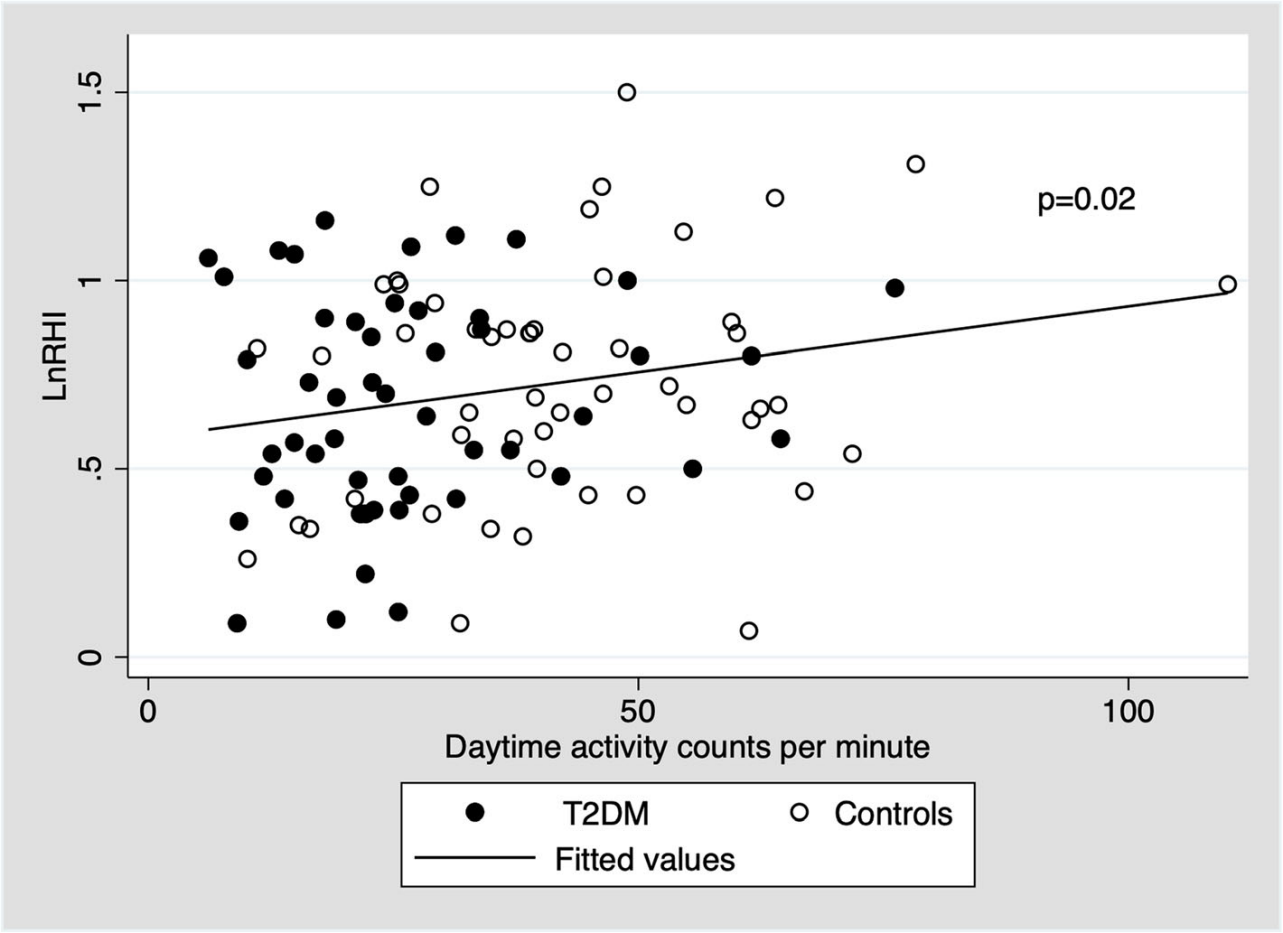
Daytime physical activity at baseline and endothelial function (lnRHI) after 5 years

Daytime physical activity at baseline was associated with lnRHI at follow-up for both patients with type 2 diabetes and controls, see Fig. 1. This association remained statistically significant when adjusted for age, sex and diabetes, see Table 2. No interaction with diabetes status was observed (*p* = 0.79). Further adjustment for smoking status, 24-h blood pressure, LDL-cholesterol, urinary albumin:creatinine ratio and BMI did not attenuate the association. To convert this finding to a clinically meaningful association, we calculated the percentual change of RHI that equals a 1 SD change in cpm. We found that an increase of 1 SD in daytime physical activity corresponded to a 6.7 % increase in RHI (95 % confidence interval: 1.1;12.5 %, *p* = 0.02).

**Table 2:** Univariate and multivariate linear regression models used to test for association between daytime physical activity and endothelial function

Linear regression model	Beta (95 %-CI)	*p*-value
Crude	0.003 (0.0006;0.006)	0.02
Model 1	0.003 (0.0005;0.006)	0.02
Model 2	0.003 (0.0002;0.006)	0.04

Beta: Change in lnRHI per daytime activity count

Model 1: Adjusted for age, sex and diabetes

Model 2: Adjusted for age, sex, diabetes, smoking status, 24-h BP, LDL-cholesterol, UACR and BMI

We found no difference in lnRHI between patients with type 2 diabetes and controls (0.67 ± 0.29 vs. 0.73 ± 0.31, *p* = 0.28). Patients with type 2 diabetes had significantly higher BMI compared to controls. Body mass index was, however, not associated with lnRHI in multivariate regression (*p* = 0.30).

## Discussion

As previously reported for this cohort, daytime physical activity was significantly lower for patients with type 2 diabetes compared to controls [28]. In the present study, we evaluated the long-term association between physical activity and endothelial function in patients with type 2 diabetes and non-diabetic controls. The main finding was that baseline physical activity was associated with endothelial function after 5 years follow-up in patients with type 2 diabetes and controls.

This expands on previous short-term studies evaluating the relationship between exercise and endothelial function. A recent meta-analysis reported improved endothelial function assessed by FMD in patients with type 2 diabetes following exercise interventions [29]. Importantly, the studies included in this meta-analysis only evaluated the effect of short-term interventions of typically 8–12 weeks. Prospective data on the long-term effect of physical activity on endothelial function are scarce. However, our results are also in line with two previous long-term observational studies demonstrating a positive association between physical activity and endothelial function in different study populations [30, 31]. In contrast to our study, these studies relied on self-reported physical activity. In a cohort with a 25-year observation period of healthy middle-aged men, Kwaśniewska et al. found that high levels of self-reported lifetime physical activity were associated with better endothelial function assessed by PAT [30]. Pahkala et al. demonstrated that increased self-reported physical activity was associated with improved endothelial function assessed by FMD in adolescents during 5 years of observation [31]. Until now, prospective data concerning the long-term association between physical activity and endothelial function assessed by PAT in patients with type 2 diabetes have been lacking.

We found no interaction with diabetes with regard to the association between physical activity and endothelial function. A possible explanation for this finding may be that the participants with type 2 diabetes had a short duration of diabetes, were well regulated with regard to glycemia, and, due to more intensive treatment, had fine blood pressure and blood lipid control.

Likewise, no difference in endothelial function between the two groups was observed. Previous cross-sectional studies have reported diverging results. Lower RHI in diabetes patients were reported in some studies [16–21], whereas other studies reported comparable RHI in patients with versus without diabetes [22–25]. Compared to our study population, the studies reporting lower RHI in diabetes patients were characterized by poorer glycemic control (HbA_1C_ = 77 ± 13 mmol/mol) [18], longer diabetes duration (14 ± 9 years) [20], or a higher proportion of patients with a history of CVD (46 %) [21]. The association with CVD was specifically evaluated in two studies. Lower RHI was reported in patients with type 2 diabetes with CVD compared to non-diabetic subjects without CVD. Conversely, no difference was observed in RHI between patients with diabetes and non-diabetic subjects with CVD [24, 25]. Also, Aragones et al. found comparable levels of RHI in patients with diabetes compared to non-diabetic subjects at intermediate risk of CVD [22]. Thus, a possible explanation for our neutral finding may be that the participants with type 2 diabetes had a short duration of diabetes, good glycemic, blood pressure and lipid control. On the other hand, patients with type 2 diabetes had significantly higher BMI compared to controls. Adjustment for BMI in multivariate regression did, however, not change the results. Moreover, as previous studies have reported positive effects of both antihypertensives and statins on endothelial function, this may have attenuated potential differences in lnRHI between the groups [37, 38]. Finally, we found numerically lower LnRHI in patients with type 2 diabetes compared to controls, and the lack of statistical significance could be due to a type II error.

Low levels of physical activity have been associated with increased risk of CVD [39], and induction of endothelial dysfunction has been suggested as a causal mechamism [40, 41]. The beneficial effects of physical activity on endothelial function are thought to be associated with the repeated shear stress stimulation of the vessels during exercise, which leads homeostatic changes with increased nitric oxide bioavailability [42]. Ultimately, these changes induce arterial adaptations that may influence the risk of cardiovascular disease.

A limitation to this study is that endothelial function was only assessed after 5 years of follow-up, hence baseline differences or temporal changes in endothelial functions could not be assessed. In addition, a number of participants did not attend the follow-up visit, which could have biased the results. However, baseline characteristics between patients with vs. without follow-up data were comparable and the bias from dropouts and missing data are most likely of little significance. Strengths of the study include a very well characterized study population. Also, the patients with type 2 diabetes were treated according to current guidelines with good risk factor control. Moreover, as opposed to previous prospective studies, physical activity was objectively measured using accelerometery. Thus, the potential for recall bias was not an issue in this regard.

## Conclusions

In conclusion, daytime physical activity was significantly lower in patients with type 2 diabetes and associated with endothelial function after 5 years follow-up in both patients with type 2 diabetes and controls.

## Supplementary Information

**Additional file 1: Supplemental Table 1.** Baseline characteristics of participants with type 2 diabetes that attended follow-up (group I) vs. patients with type 2 diabetes that did not attend follow-up or had missing data (group II). **Supplemental Table 2.** Baseline characteristics of control subjects that attended follow-up (group I) vs. control subjects that did not attend follow-up or had missing data (group II).

## Data Availability

All data generated and/or analyzed in the present study are available from the corresponding author on request.

## Abbreviations

BP: Blood pressure
BMI: Body mass index
CI: Confidence interval
CV: Cardiovascular
CVD: Cardiovascular disease
FMD: Flow-mediated vasodilation
PAT: Peripheral arterial tonometry
RHI: Reactive Hyperemia Index
lnRHI: Natural logarithm Reactive Hyperemia Index
Cpm: Counts per minute
UACR: Urinary albumin:creatinine ratio
