# Supplementary material for: Low physical activity is associated with impaired endothelial function in patients with type 2 diabetes and controls after 5 years of follow-up
Source: BMC Endocr Disord. 2021 Sep 18;21:189. doi: 10.1186/s12902-021-00857-9 (PMC8449475; doi:10.1186/s12902-021-00857-9)
Supplement: Supplementary file 1 — Additional file 1: Supplemental Table 1. Baseline characteristics of participants with type 2 diabetes that attended follow-up (group I) vs. patients with type 2 diabetes that did not attend follow-up or had missing data (group II). Supplemental Table 2. Baseline characteristics of control subjects that attended follow-up (group I) vs. control subjects that did not attend follow-up or had missing data (group II). [file 12902_2021_857_MOESM1_ESM.docx]

Supplemental Table 1. Baseline characteristics of participants with type 2 diabetes that attended follow-up (group I) vs. patients with type 2 diabetes that did not attend follow-up or had missing data (group II).

| **Characteristic** | **Group I (*n*=51)** | **Group II (*n*=49)** | | ***p*-value** |
| --- | --- | --- | --- | --- |
| *Clinical* |  |  |  | |
| Age at baseline, years | 58.9 ± 10.0 | 58.0 ± 9.7 | 0.67 | |
| Male sex, n (%) | 30 (59) | 22 (45) | 0.16 | |
| Diabetes duration at follow-up, years | 7.9 (6.43;9.1) | 6.6 (6.3;8.5)^a^ | 0.26 | |
| 24h blood pressure, mmHg |  |  |  | |
| Systolic | 126 ± 12^b^ | 125 ± 10^c^ | 0.55 | |
| Diastolic | 75 ± 7^b^ | 74 ± 8^c^ | 0.61 | |
| Office blood pressure, mmHg |  |  |  | |
| Systolic | 127 ± 11^d^ | 124 ± 12^e^ | 0.92 | |
| Diastolic | 80 ± 8^d^ | 78 ± 8^e^ | 0.82 | |
| Heart rate (beats/minute) | 65 ± 10^b^ | 67 ± 10^f^ | 0.18 | |
| BMI, kg/m^2^ | 29.9 ± 4.9 | 30.2 ± 4.7 | 0.39 | |
| Smoking status |  | ^g^ | 0.51 | |
| Current, *n* (%) | 9 (18) | 12 (25) |  | |
| Former *n* (%) | 21 (41) | 15 (31) |  | |
| Never, *n* (%) | 21 (42) | 21 (44) |  | |
| Previous CVD, *n* (%) | 11 (22) | 8 (16) | 0.50 | |
| *Biochemical* |  |  |  | |
| HbA_1C._ mmol/mol | 48 ± 8 | 48 ± 6 | 0.27 | |
| HbA_1C_, (%) | 6.5 ± 0.7 | 6.6 ± 0.6 | 0.27 | |
| Total cholesterol, mmol/L | 4.3 ± 0.8 | 4.5 ± 0.9 | 0.14 | |
| HDL-C, mmol/L | 1.4 ± 0.3 | 1.4 ± 0.3 | 0.67 | |
| LDL-C, mmol/L | 2.2 ± 0.7^d^ | 2.5 ± 0.7 | 0.02 | |
| Triglycerides, mmol/L | 1.6 (1.0;2.2) | 1.3 (1.1;1.7) | 0.14 | |
| UACR, mg/g | 0.46 (0.28;1.03) | 0.37 (0.27;0.72) | 0.53 | |
| *Medication* |  |  |  | |
| Antihypertensive treatment, *n* (%) | 34 (67) | 29 (60) | 0.53 | |
| Diabetes treatment |  |  |  | |
| Metformin, *n* (%) | 32 (63) | 30 (63) | 0.98 | |
| Sulfonylureas, *n* (%) | 6 (12) | 7 (15) | 0.68 | |
| GLP-1 agonist, *n* (%) | 0 (0) | 2 (4) | 0.14 | |
| DPP4 inhibitor, *n* (%) | 2 (4) | 1 (2) | 0.59 | |
| Insulin, *n* (%) | 4 (8) | 4 (8) | 0.95 | |
| Acetylsalicylic acid, *n* (%) | 32 (63) | 29 (60) | 0.81 | |
| Statin, *n* (%) | 38 (75) | 38 (79) | 0.58 | |
| ^a^ *n* = 12, ^b^ *n* = 50, ^c^ *n* = 46, ^d^ *n =* 49, ^e^ *n =* 47, ^f^ *n* = 50, ^g^ *n* = 48 | | | | |

Supplemental Table 2. Baseline characteristics of control subjects that attended follow-up (group I) vs. control subjects that did not attend follow-up or had missing data (group II).

| **Characteristic** | **Group I (*n*=53)** | **Group II (*n*=47)** | ***p*-value** |
| --- | --- | --- | --- |
| *Clinical* |  |  |  |
| Age at baseline, years | 57.8 ± 10.0 | 58.9 ± 9.6 | 0.70 |
| Male sex, n (%) | 28 (53) | 24 (51) | 0.86 |
| 24h blood pressure, mmHg |  |  |  |
| Systolic | 124 ± 11^a^ | 126 ± 14^b^ | 0.16 |
| Diastolic | 75 ± 7^a^ | 77 ± 8^b^ | 0.07 |
| Office blood pressure, mmHg |  |  |  |
| Systolic | 131 ± 15 | 133 ± 16 | 0.27 |
| Diastolic | 83 ± 10 | 85 ± 11 | 0.10 |
| Heart rate (beats/minute) | 60 ± 9 | 64 ± 12^c^ | 0.03 |
| BMI, kg/m^2^ | 26.3 ± 3.8 | 25.9 ± 4.3 | 0.67 |
| Smoking status |  |  | 0.08 |
| Current, *n* (%) | 7 (13) | 14 (30) |  |
| Former *n* (%) | 17 (32) | 16 (34) |  |
| Never, *n* (%) | 29 (55) | 17 (36) |  |
| Previous CVD, *n* (%) | 6 (11) | 7 (15) | 0.60 |
| *Biochemical* |  |  |  |
| HbA_1C._ mmol/mol | 38 ± 4 | 38 ± 4 | 0.86 |
| HbA_1C_, (%) | 5.7 ± 0.3 | 5.6 ± 0.4 | 0.86 |
| Total cholesterol, mmol/L | 5.7 ± 1.0 | 5.7 ± 1.0 | 0.54 |
| HDL-C, mmol/L | 1.7 ± 0.6 | 1.7 ± 0.5 | 0.65 |
| LDL-C, mmol/L | 3.3 ± 1.0 | 3.4 ± 0.9^c^ | 0.41 |
| Triglycerides, mmol/L | 1.2 (0.9;1.6) | 1.2 (0.8;1.5) | 0.89 |
| UACR, mg/g | 0.23 (0.17;0.34) | 0.30 (0.17;0.50) | 0.17 |
| *Medication* |  |  |  |
| Antihypertensive treatment, *n* (%) | 15 (28) | 10 (21) | 0.42 |
| Acetylsalicylic acid, *n* (%) | 3 (6) | 5 (11) | 0.36 |
| Statin, *n* (%) | 10 (19) | 8 (17) | 0.81 |
| ^a^ *n* = 52, ^b^ *n* = 46, ^c^ *n* = 46 | | | |
